# First principles calculations of the thermodynamic stability of Ba, Zr, and O vacancies in BaZrO_3_

**DOI:** 10.1039/c9ra07978e

**Published:** 2019-10-23

**Authors:** N. Raja, D. Murali, S. V. M. Satyanarayana, M. Posselt

**Affiliations:** Department of Physics, Pondicherry University Puducherry 605 014 India; Indian Institute of Information Technology Design and Manufacturing Kurnool Andhra Pradesh 518002 India; Helmholtz-Zentrum Dresden-Rossendorf, Institute of Ion Beam Physics and Materials Research Bautzner Landstraβe 400 01328 Dresden Germany m.posselt@hzdr.de

## Abstract

The temperature dependence of the stability of bulk BaZrO_3_ (BZO) and of the vacancies in this material are investigated by considering phonon contributions to the free energy. The stability diagram of BZO is determined for different chemical environments. With increasing temperature the stability region becomes smaller which is particularly caused by the strong temperature dependence of the chemical potential of gaseous oxygen. The free formation energy of Ba, Zr, and O vacancies in BZO is calculated for all possible charge states and for different atomic reservoirs. While the free formation energy of Zr vacancies is strongly influenced by temperature a weaker dependence is found for Ba and O vacancies. This also has an effect on the charge transition levels at different temperatures. The present results demonstrate that O poor reservoir conditions and a Fermi level close to the valence band maximum favour a high concentration of doubly positively charged O vacancies which is a prerequisite to get a large number of protonic defects and good proton conductivity. In such a chemical environment the number of Ba and Zr vacancies is low so that Ba and Zr deficiencies are not an important issue and BZO remains sufficiently stable.

## Abstract 2

The temperature dependence of the free formation energy of Ba, Zr and O vacancies in BaZrO_3_ is investigated for all possible charge states and atomic reservoir conditions.

## Graphical abstract

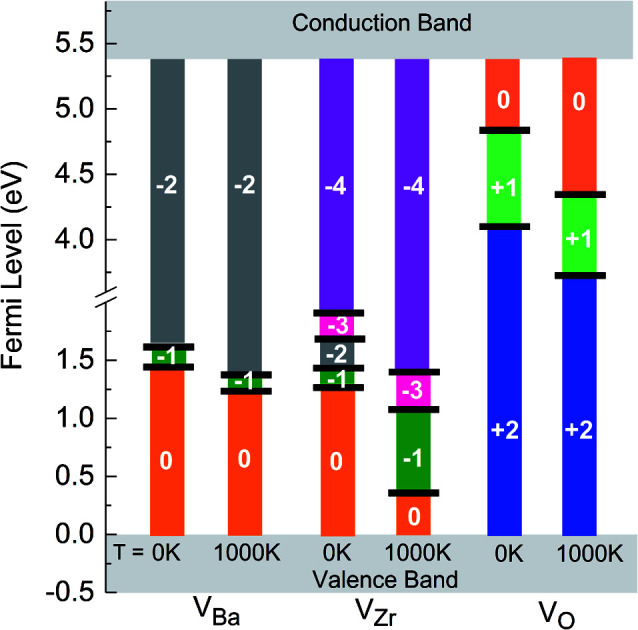

## Introduction

BaZrO_3_ (BZO) is a prototypical perovskite-structured material with oxygen-ion and proton conducting properties.^1–3^ BZO is a candidate material for various applications such as solid oxide fuel cells, gas sensors, and hydrogen pumps.^4–8^ It shows good chemical and thermodynamic stability even at very high temperature.^9,10^ BZO becomes proton conducting after exposure to humid conditions.^10,11^ The water from the gas phase dissociates into a hydroxide ion and a proton, the hydroxide ion fills a doubly positively charged oxygen vacancy, and the proton forms a covalent bond with an oxygen atom of the perovskite lattice. Finally, two positively charged protonic defects are formed. In Kröger–Vink notation this reaction is given as^11^1
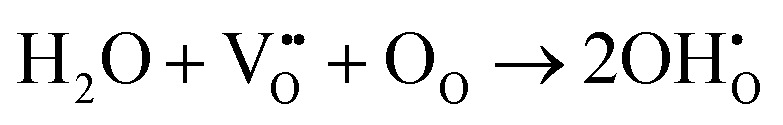


Oxygen vacancy concentration in BZO can be increased by various techniques like strain engineering, doping, *etc.* Doping of BZO with lower valence cations such as Y can essentially improve the proton conductivity.^12–14^

First principles Density Functional Theory (DFT) calculations can contribute to the understanding of the promising properties of BZO. Recently, the stability of bulk BZO and of Ba, Zr, and O vacancies at ground state has been extensively studied by DFT methods.^15^ Since solid-state proton conductors such as BZO operate at relatively high temperatures, it is also very important to investigate the thermodynamic stability of the differently charged vacancy defects in dependence on the chemical environment. Sundell *et al.*^16^ used DFT to study the neutral and fully charged Ba, Zr and O vacancies in the ground state and determined the temperature dependence of the formation of neutral and doubly positively charged O by thermodynamic modelling. In a previous DFT work we found that at 1000 K phonon contributions increase the migration barrier of the doubly positively charged oxygen vacancy by 0.35 eV.^17^ Bjørheim *et al.* investigated the neutral and the doubly positively charged state of the O vacancy at finite temperatures and found that near the conduction band minimum the phonon contribution decreases the free formation energy of the neutral vacancy by around 0.8 eV at 1000 K, but does not alter the free formation energy of the doubly positively charged O vacancy.^18^ However, in this study only the O poor chemical environment was considered. The absorption of CO_2_ at high temperature was investigated in pristine as well as Y doped BZO by Polfus *et al.* and the role of Ba, Zr, and O vacancies was discussed.^19^ Recent experimental studies clearly indicate that considerable Ba deficiency may occur at high temperatures.^20^ This leads to a drastic decreases of proton conductivity^21^ and material stability.^22^ Cation vacancy defects influence the site occupancy of foreign atoms and dopants.^23,24^ In this context, it is very important to determine the properties of the Ba vacancy at high temperatures. Also the Zr vacancies are responsible for the electrochemical properties and the thermodynamic stability of BZO. It is therefore important to study the temperature dependence of the free formation energy of all the possible vacancies in BZO, for different charge states and various atomic reservoir conditions. To the best of our knowledge, such comprehensive investigations have not been performed yet.

In this work, a systematic first-principles study on the thermodynamic stability of Ba, Zr, and O vacancies in BZO is performed. In the first part, the thermodynamic stability of BZO is determined in dependence on temperature and on the chemical potentials of atomic reservoirs, which are in equilibrium with BZO, *i.e.* on different conditions of BZO synthesis. The free formation energy of neutral and charged Ba, Zr, and O vacancies and the corresponding charge transition levels are calculated in the second part, with particular emphasis on the influence of Fermi level, chemical environment, and temperature. In this manner, conditions for the preferential formation of doubly positively charged O vacancies and for the limitation of the number of Ba and Zr vacancies can be identified. These conditions are important prerequisites for achieving optimum proton conductivity in BZO.

## Computational details

DFT calculations were performed using the Vienna *Ab Initio* Simulation Package (VASP) which employs plane wave basis sets.^25,26^ The pseudopotentials are based on the projector augmented wave (PAW) method with exchange and correlation effects described by the generalized gradient approximation (GGA-RPBE).^27^ Brillouin zone sampling is done using the Monkhorst–Pack scheme.^28^ Ground state calculations are performed in a 40 atom supercell with 4 × 4 × 4 *k*-points. A plane wave basis cutoff of 500 eV is chosen for all calculations. Atomic positions as well as cell shape and volume are allowed to relax until the total stress/pressure on the supercell becomes zero. In order to obtain accurate final equilibrium geometries, thresholds for total energy change and total force acting on each atom are chosen as 10^−8^ eV and 10^−6^ eV Å^−1^, respectively. To model charged defects, the appropriate number of electrons is removed from or added to the supercell and charge compensation is performed by a homogeneous jellium background charge. For example, in the case of the doubly positively charged oxygen vacancy two electrons are removed from the overall supercell. In the VASP code an approach similar to Makov and Payne is used in order to correct for the artificial interactions between charged defects.^29^

Table 1 demonstrates that the above mentioned choice of VASP input data yields reasonable values for ground state structural properties of solid Ba, Zr, BaO, ZrO_2_, and BZO, and the O_2_ molecule.

**Table 1:** Calculated values of the ground state structural properties of Ba, Zr, O, BaO, ZrO_2_ and BZO, compared with theoretical and experimental data from literature. *a*: lattice constant, *c*/*a*: axial ratio and *r*_0_: bond length

	This work	Theory	Experiment
Ba (BCC)			
*a* (Å)	5.039	4.770 (ref. 30)	5.020 (ref. 31)
Zr (HCP)			
*a* (Å)	3.220	3.229 (ref. 32)	3.232 (ref. 27)
*c*/*a*	1.732	1.600 (ref. 25)	1.592 (ref. 27)
O (dimer)			
*r*_0_ (Å)	1.215	1.22 (ref. 25)	1.21 (ref. 26)
BaO (cesium chloride)			
*a* (Å)	5.515	5.576 (ref. 15)	5.539 (ref. 28)
ZrO_2_ (monoclinic)			
*a* (Å)	5.194	5.217 (ref. 15)	5.151 (ref. 29)
*c*/*a*	1.039	1.034 (ref. 15)	1.032 (ref. 29)
BaZrO_3_ (cubic)			
*a* (Å)	4.269	4.208 (ref. 15)	4.192 (ref. 30)

The frozen phonon approach and the harmonic approximation^33,34^ are applied to determine the vibrational frequencies which are used to investigate the effect of temperature on the materials properties. In these calculations, ground state atomic positions are slightly displaced (by 0.015 Å) in positive and negative *x*-, *y*- and *z*-directions to obtain the respective force derivatives for the dynamical matrix. The symmetry of the underlying supercell is used to reduce the number of atomic displacements and thus computational costs. The diagonalization of the dynamical matrix yields all vibrational frequencies *ω*_i_ of the supercell. All the calculations are performed for the gamma point in the space of the phonon wave vectors. At a given temperature *T* the phonon or vibrational contribution *G*^vib^(*T*) to the Gibbs free energy of a system of N atoms is defined by2
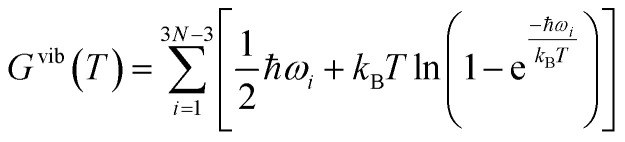
where *ħ* and *k*_B_ are the Planck's and Boltzmann's constants, respectively.

### Gibbs free energy of formation of BZO, BaO and ZrO_2_

At ground state the energy of formation of a compound is defined as the difference between the energy of the compound and the sum of energies of the elemental constituents. In the case of BZO, BaO and ZrO_2_ the energy of formation per formula unit is given by:^35–38^3
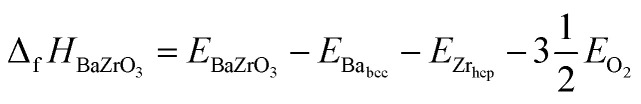
4
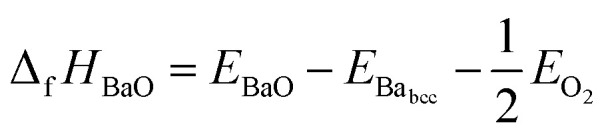

5 Δ_f_*H*_ZrO_2__ = *E*_ZrO_2__ − *E*_Zr_hcp__ − *E*_O_2__

where, *E*_BaZrO_3__, *E*_BaO_, *E*_ZrO_2__, *E*_Ba_bcc__, *E*_Zr_hcp__ and *E*_O_2__ are the ground state energies of BZO, BaO, ZrO_2_, Ba, Zr, and the O_2_ molecule, respectively.

While DFT is able to determine rather precise values for the ground state energies of BZO, BaO, ZrO_2_, Ba, and Zr, this method yields poor data for the ground state energy of the O_2_ molecule. Wang *et al.*^39^ attributed this to the over binding caused by treatment of exchange and correlation effects by the GGA. These authors also found that this issue leads to a difference of about 1.36 eV (per O_2_ molecule) between theoretical and experimental data for the oxidation energy of many metal oxides. Therefore, in the present work this value is added to the DFT value for *E*_O_2__ which corresponds to the procedure proposed by Wang *et al.*^39^ This correction was also used by Bjørheim *et al.* in the calculation of the free formation energy of the oxygen vacancy.^18^

The data obtained for the formation energy of BZO, BaO and ZrO_2_ are −18.46 eV, −4.98 eV and −11.03 eV, respectively. These data are slightly different to previously reported ground state DFT results of −16.73 eV (ref. 16) and −17.82 eV (ref. 15) for BZO, −5.17 eV (ref. 16) and −5.55 eV (ref. 15) for BaO, and −10.25 eV (ref. 16) and −10.27 eV (ref. 15) for ZrO_2_, since in these papers the O_2_ correction was not taken into account.

**Fig. 1:**
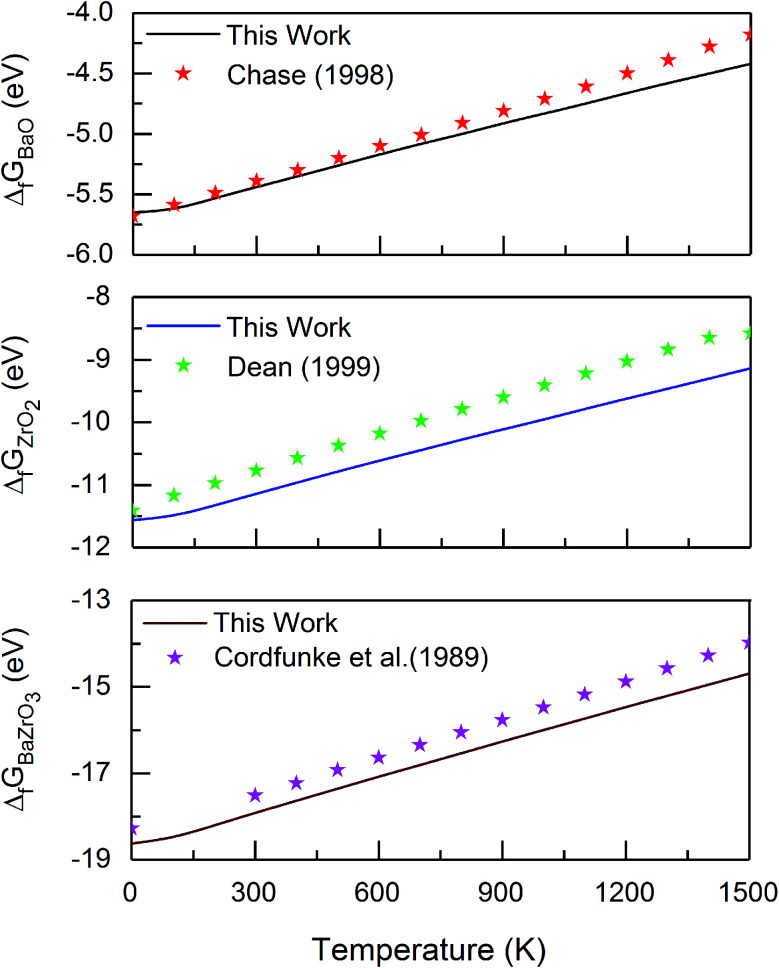
Gibbs free energy of formation of BaO, ZrO_2_ and BZO per formula unit, in comparison with reported data obtained from experiments and thermodynamic modelling.^40–42^

The vibrational contribution to the Gibbs free energy of formation per formula unit is given by6
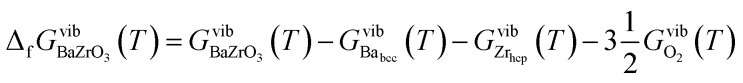
7
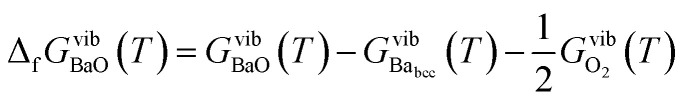
8
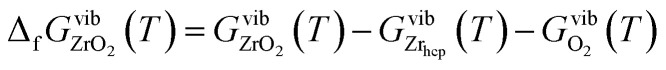
where *G*^vib^ are the respective vibrational free energies at temperature *T*, see eqn (2). The total Gibbs free energy of formation of BZO (Δ_f_*G*_BaZrO_3__), BaO (Δ_f_*G*_BaO_), and ZrO_2_ (Δ_f_*G*_ZrO_2__), is then determined by the sum of the corresponding quantities in eqn (3)–(8). The temperature dependence of the (total) Gibbs free energy of formation is presented in Fig. 1 along with thermochemical reference data.^40–42^

For all three compounds the absolute value of the Gibbs free energy of formation decreases if the temperature increases. There is a very good agreement between calculated and experimental data at lower temperatures. The discrepancy arising at high temperatures may be explained by anharmonic effects that are not taken into account in the calculation. The contribution of zero-point vibrations to the Gibbs free energy of formation is −0.16 eV, −0.67 eV and −0.53 eV for BZO, BaO and ZrO_2_ respectively. Therefore, at 0 K the Gibbs free energy of formation is −18.62 eV (BZO), −5.65 eV (BaO), and −11.56 eV (ZrO_2_), which is in good agreement with the thermochemical reference data of −18.27 eV (ref. 40), −5.68 eV (ref. 41), and −11.41 eV (ref. 42) for BZO, BaO and ZrO_2_, respectively. This also demonstrates that the correction of the ground state energy *E*_O_2__ (see above) leads to consistent results.

The Gibbs free energy of formation of BZO (per formula unit) from BaO and ZrO_2_ is defined similarly to eqn (3)–(8)9
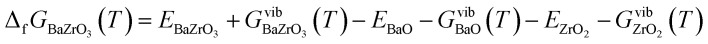


**Fig. 2:**
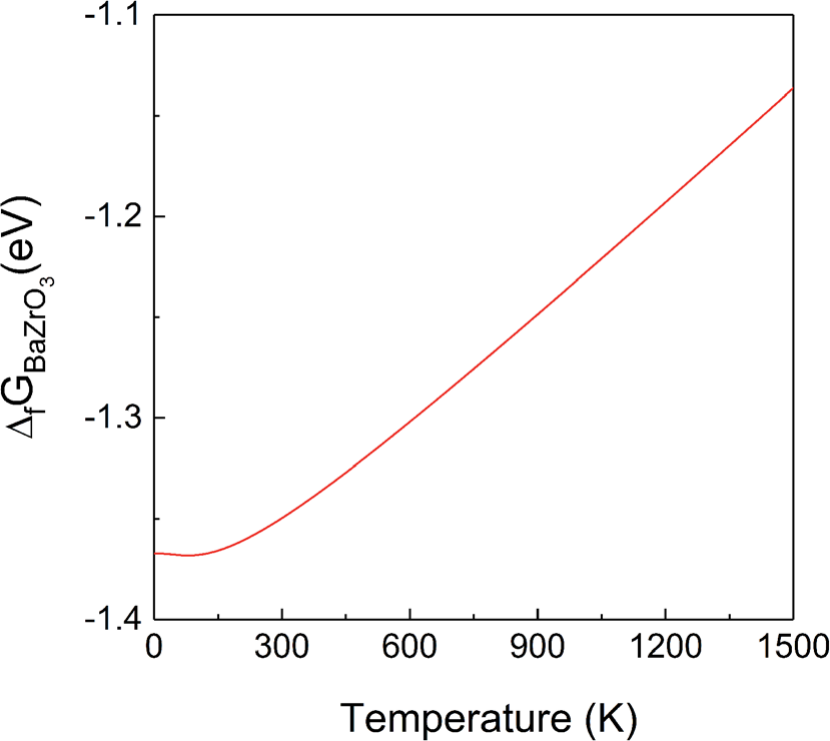
Gibbs free energy of formation of BZO (per formula unit) from BaO and ZrO_2_ as a function of temperature.

Fig. 2 shows that the absolute value of this quantity decreases with temperature, *i.e.* the probability of dissociation of BZO into BaO and ZrO_2_ increases. This trend is in agreement with experimental results.^43^ The quantitative difference at higher temperature may be due to anharmonic effects, which are not considered in the present work. The difference between the ground state value of Δ_f_*G*_BaZrO_3__ and that for 300 K is −1.34 eV, which is in good agreement with the experimental values of −1.33 eV and −1.19 eV at 300 K.^43,44^

Fig. 1 and 2 clearly demonstrate the importance of vibrational effects on the thermodynamic stability of BZO, BaO and ZrO_2_.

### Chemical stability of BZO

The chemical stability of BZO can be determined from the constraints that give valid chemical potentials of atomic reservoirs in equilibrium with BZO. The chemical potential of an atom species i is defined as

10 *μ*_i_ = *μ*_i_^0^ + Δ*μ*_i_

Here, the quantities *μ*_i_^0^ are the chemical potentials of Ba, Zr, and O atoms in their standard solid (Ba, Zr) and gaseous (oxygen) reference states.

The stability of BZO is investigated by imposing constraints on Δ*μ*_i_. For example, the set of constraints that do not allow BZO to decompose into elements Ba, Zr and O_2_ is given by

11 Δ*μ*_Ba_ ≤ 0, Δ*μ*_Zr_ ≤ 0 and Δ*μ*_O_ ≤ 0

Further, constraints that do not allow BZO to decompose into binary oxides BaO and ZrO_2_ are governed by

12 Δ_f_*G*_BaO_ ≥ Δ*μ*_Ba_ + Δ*μ*_O_

13 Δ_f_*G*_ZrO_2__ ≥ Δ*μ*_Zr_ + 2Δ*μ*_O_

These constraints together with the following equation allow us to compute the stability diagram, where the suitable chemical environment for the formation of BZO can be determined.

14 Δ_f_*G*_BaZrO_3__ = Δ*μ*_Ba_ + Δ*μ*_Zr_ + 3Δ*μ*_O_

**Fig. 3:**
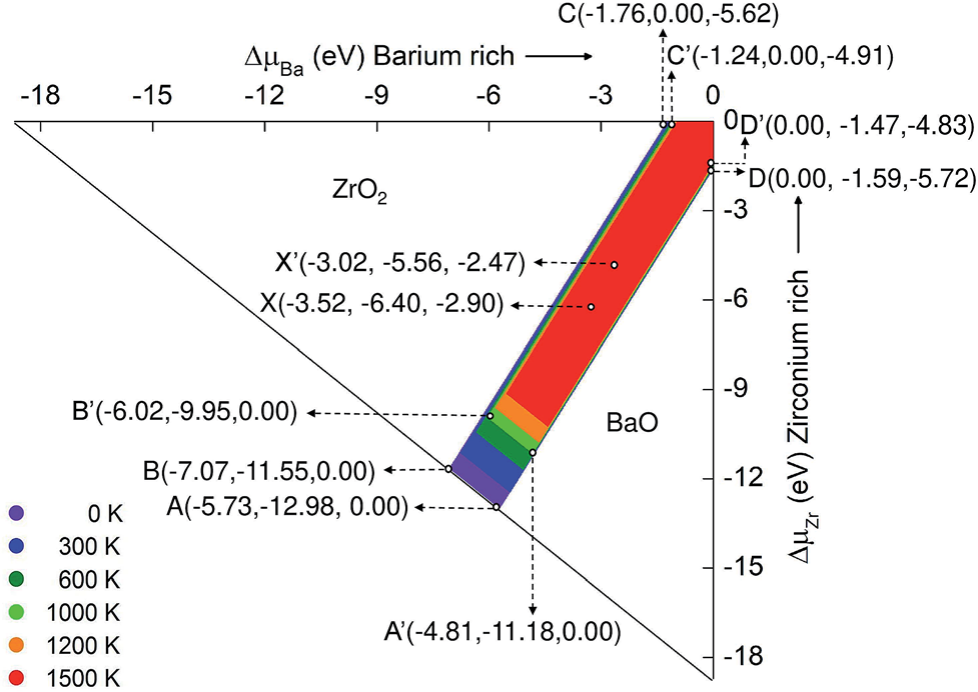
The area of chemical stability of BZO for different temperature, from 0 K to 1500 K. At 0 K the points A, B, C, 0 and D are the corners of the stability region, whereas at 1000 K BZO is stable within the area limited by A′, B′, C′, 0, and D′. The first, second, and third coordinates denote the values of Δ*μ*_i_ for Ba, Zr, and O, respectively.

Fig. 3 represents the stability diagram of BZO with the competing binary oxides BaO and ZrO_2_. Points A, B, C, D and X represent five different chemical potential conditions at 0 K. These conditions are obtained from eqn (10)–(14) and using the corresponding values for the Gibbs free energy of formation at 0 K. Points A and B correspond to O rich conditions with the chemical potential value Δ*μ*_O_ = 0 eV. At point C the environment is Zr rich, at point D, Ba rich. The condition of extreme reduction can be obtained at point C, where the chemical potential of oxygen is most negative: Δ*μ*_O_ = −5.78 eV. Point X is in the intermediate stability region of BZO between oxidation and reduction conditions.

Fig. 3 can be compared with the results obtained by Abbas *et al.*^15^ They calculated the stability diagram for the ground state using all electron calculation (WIEN2k code) and did not include the O_2_ correction (see above). Comparison of our ground state chemical potential values of Ba, Zr, and O with the literature data may helpful to understand the significance of O_2_ correction. For example, at point C, O poor/Zr rich conditions, the atomic chemical potential values for the ground state obtained from our calculation is −1.96 (Δ*μ*_Ba_), 0.00 (Δ*μ*_Zr_) and −5.50 (Δ*μ*_O_), which is slightly different from the literature values of −2.423 (ref. 15), 0.000 (ref. 15) and −5.134 (ref. 15) for Δ*μ*_Ba_, Δ*μ*_Zr_, and Δ*μ*_O_ respectively. The deviations are mainly due to the inclusion of the O_2_ correction, but certainly also due to the use of a pseudopotential in the VASP calculations. At other points of the stability diagram similar differences between our ground state values of the chemical potential and the corresponding data of Abbas *et al.*^15^ occur.

As the temperature increases, the region of chemical stability of BZO becomes smaller. This is due to the temperature dependence of the Gibbs free energies of formation Δ_f_*G*_BaZrO_3__, Δ_f_*G*_BaO_, and Δ_f_*G*_ZrO_2__. At 1000 K the stability region of BZO is limited by the points A′, B′, C′, 0, and D′. At points D′ and C′ the chemical potential of oxygen is −4.83 eV and −4.98 eV, respectively. This drastic change compared to 0 K is due to the high entropy of the gaseous O_2_ molecule, which leads to a relatively fast decrease of the absolute value of the corresponding free energy if temperature increases. The strong temperature dependence of Δ*μ*_O_ is also the cause for the loss of the stability of BZO as well as BaO and ZrO_2_ by the release of oxygen, which occurs in regions that correspond to strong oxidation conditions at 0 K. Point X′ in the intermediate stability region is obtained by shifting point X towards point 0, by a distance corresponding to the difference between A and A′ or B and B′. On the other hand, the boundaries between the stability regions of BZO and BaO and those of BZO and ZrO_2_ change relatively weakly if the temperature is increased.

It should be mentioned that the relation between the chemical potential *μ*_i_ and the atomic concentration *C*_i_ is given by15
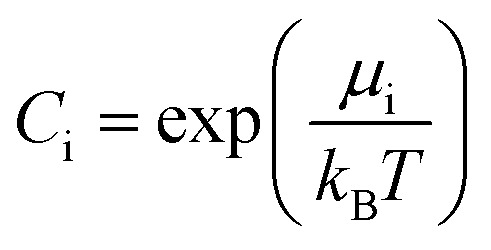


The partial oxygen pressure *p*_O_2__ can be obtained from the chemical potential *μ*_O_ using16
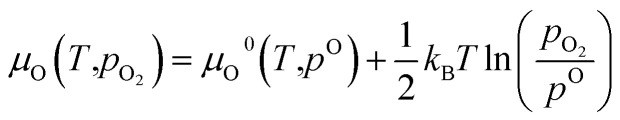
where *p*^O^ denotes the standard or reference pressure. The quantity *μ*_O_^0^ is equal to the half of the free energy of the O_2_ molecule at standard pressure (see *e.g.*ref. 18). In this manner, the stability of BZO may be also represented in dependence on concentration and partial pressure.

### Gibbs free formation energy of Ba, Zr and O vacancies in BZO

In solid compounds like BZO, BaO, or ZrO_2_ the vacancy formation is influenced by the chemical potential of the individual atom species, which constitute the material. Furthermore, the vacancy may have different charge states. The ground state formation energy of a vacancy in a charge state *q* is defined as (see *e.g.*ref. 15)

17 Δ*E*_f_(*V*_i_^*q*^) = *E*_tot_(*V*_i_^*q*^) − *E*_tot_(bulk) + *q*(*E*_VBM_ + *E*_f_) + *μ*_i_

where *E*_tot_(*V*_i_^*q*^) and *E*_tot_(bulk) are the total energy of the supercell with and without the vacancy, respectively. *μ*_i_ is the ground state chemical potential of the atom which was removed in order to form the vacancy. *E*_VBM_ is the valence band maximum and *E*_F_ is the Fermi level of the system. By definition *E*_F_ is set to zero at VBM. The formation energy was calculated for different charge states of Ba, Zr and O vacancies in BZO, (Ba vacancy: 0, −1, −2; Zr vacancy: 0, −1, −2, −3, −4; O vacancy: 0, +1, +2). Our DFT calculations yield a band gap value of 3.20 eV. It is well known that the GGA used in DFT underestimates the band gap.^45^ Therefore, in this work the experimental value of 5.33 eV is used in eqn (17).^46^ It should be mentioned that the so-called over binding problem (oxygen energy see third section of this work) by the RPBE exchange-correlation functional as well as the underestimation of the band gap by this procedure might be overcome using the Heyd–Scuseria–Ernzerhof (HSE) functional^47–49^ as well as other sophisticated approaches.^50^ However, this would increase the computational costs and was therefore not considered in this work.

At temperature *T* the Gibbs free formation energy of a vacancy is defined similarly to eqn (17)

18 Δ*G*_f_(*V*_i_^*q*^,*T*) = *G*_tot_(*V*_i_^*q*^,*T*) − *G*_tot_(bulk,*T*) + *q*(*E*_VBM_ + *E*_F_) + *μ*_i_(*T*)

Where *G*_tot_(*V*_i_^*q*^,*T*) and *G*_tot_(bulk,*T*) are the total free energies of the system with and without the vacancy, respectively. These quantities correspond to the sum of the ground state energy and the respective vibrational contribution as given by eqn (2). The value of *μ*_i_(*T*) is determined according to eqn (10). It should be mentioned the temperature dependence of the difference *G*_tot_(*V*_i_^*q*^,*T*) − *G*_tot_(bulk,*T*) was also calculated by Bjørheim *et al.* for the neutral and the doubly positively charged O vacancy.^18^ Our results agree well with those of ref. 18 and 45.

**Fig. 4:**
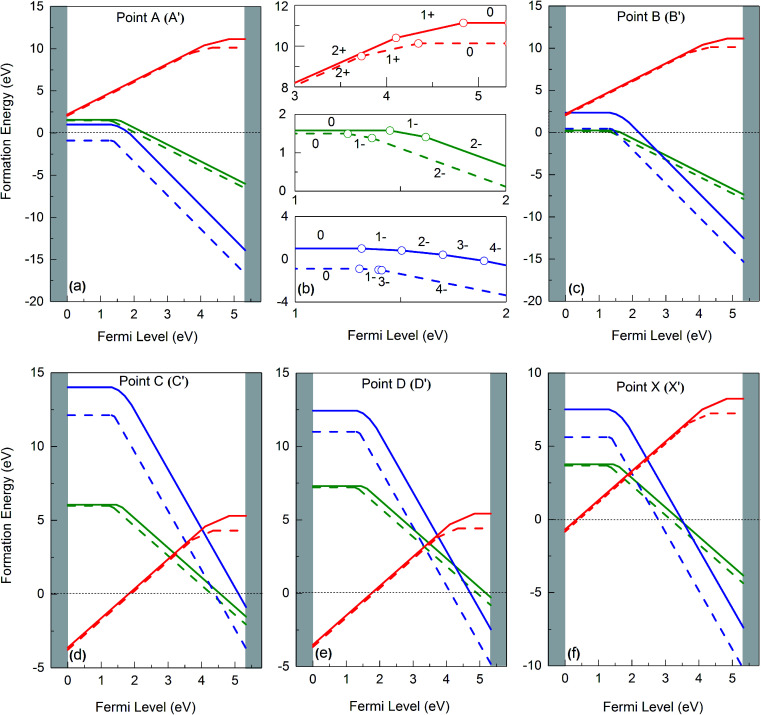
Gibbs free formation energies of Ba (green), Zr (blue) and O (red) vacancies at 0 K (solid line) and 1000 K (dashed line), as function of Fermi energy, for different chemical environments (see Fig. 3).

The solid lines in Fig. 4 depict the free formation energies of vacancies at 0 K for reservoir conditions corresponding to points A, B, C, D and X of the stability diagram (Fig. 3), whereas the dashed lines are the data for 1000 K and for points A′, B′, C′, D′, and X′. In dependence on the Fermi energy neutral or charged vacancies are energetically favored. In the case of points A and A′ the charge transitions are illustrated in detail in Fig. 4b. Because of eqn (17), the Fermi level at which the charge transitions occur is independent of reservoir conditions, but the transition level depends on temperature. It should be noticed that for a given Fermi energy the diagrams show only values for the lowest free formation energy among all the possible charge states. Close to the VBM the O vacancy has the lowest free formation energy while the free formation energy of Ba and Zr vacancies is highest. The opposite behavior is found near the conduction band minimum (CBM). The values of the free formation energy strongly depend on the reservoir conditions. At points C and C′ as well as at D and D′ (oxygen poor conditions) the most stable defect is the O vacancy in the +2 charge state if the Fermi level is closer to VBM than to CBM. For other values of *E*_F_ the negatively charged Ba and Zr vacancies are more stable. Under O rich conditions (points A and A′, B and B′) Ba and Zr vacancies are more stable than the O vacancy, for all possible values of the Fermi energy. Near VBM these vacancies are preferentially neutral, towards the CBM they are negatively charged. In the case of the Ba and the O vacancy the difference between the data obtained for 0 and 1000 K is dependent on the value of *E*_F_, in contrast to the Zr vacancy where such a dependence is not significant while the absolute value of the difference is higher.

For O poor/Zr rich conditions, *i.e.* at point C, the ground state formation energy obtained from our calculation is 5.39 eV (*V*_Ba_^0^), 14.56 (*V*_Zr_^0^), and −3.43 (*V*_O_^2+^), whereas Abbas *et al.*^15^ obtained is 5.66 eV, 16.30 eV and −1.84 eV for *V*_Ba_^0^, *V*_Zr_^0^ and *V*_O_^2+^ respectively. Again, the differences are mainly due to the O_2_ energy correction applied in this work (see above).

**Fig. 5:**
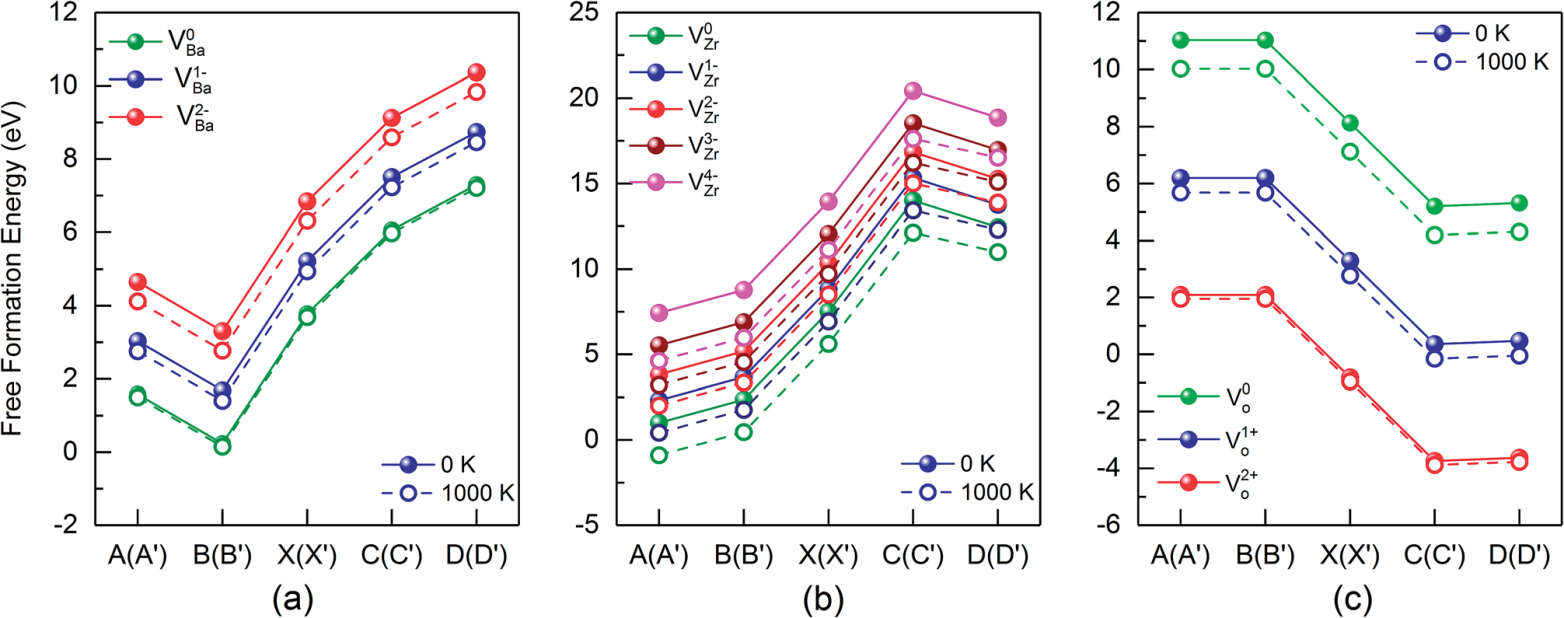
Free formation energy of Ba, Zr and O vacancies at the valence band maximum (VBM), for different points of the stability diagram (see Fig. 3). Data for 0 K and 1000 K data are shown by solid and dashed lines, respectively.

Fig. 5 summarizes the data of the free formation energy of Ba, Zr, and O vacancies at 0 and 1000 K for the case that the Fermi level is at the VBM. The stability of the Ba vacancy is highest at points B and B′ *i.e.* at O rich conditions, and the neutral charge state is most favored. On the other hand, at points D and D′, *i.e.* at Zr rich conditions, the free formation energy of the Ba vacancy is highest. The Zr vacancy shows a behavior similar to that of the Ba vacancy but has its minimum free formation energy at points A and A′. The stability of the Zr vacancy increases significantly with temperature: while at 0 K the most probable state (points A and A′) has a positive free formation energy (1.00 eV) this quantity is negative at 1000 K (−0.88 eV). Neither the Ba nor the O vacancy shows such a strong *T* dependence since in these cases the vibrational contributions to the free formation energy are lower. At and near the VBM the oxygen vacancy is most stable at points C and C′ as well as at points D and D′ whereby the doubly positively charged state is preferred. This condition favors the production of protonic defects according to reaction (1). The results depicted in Fig. 4 and 5 clearly show that the reservoir conditions at points C (C′) and D (D′) favor a high concentration of doubly positively charged O vacancies and a low concentration of Ba and Zr vacancies. This is an optimum prerequisite in order to achieve high proton conductivity in BZO while avoiding much Ba and Zr deficiencies. As shown in a previous work^17^ phonon contributions increase the migration barrier of the doubly positively charged O vacancy. This contributes to the high-temperature stability of BZO observed in experiments.

**Fig. 6:**
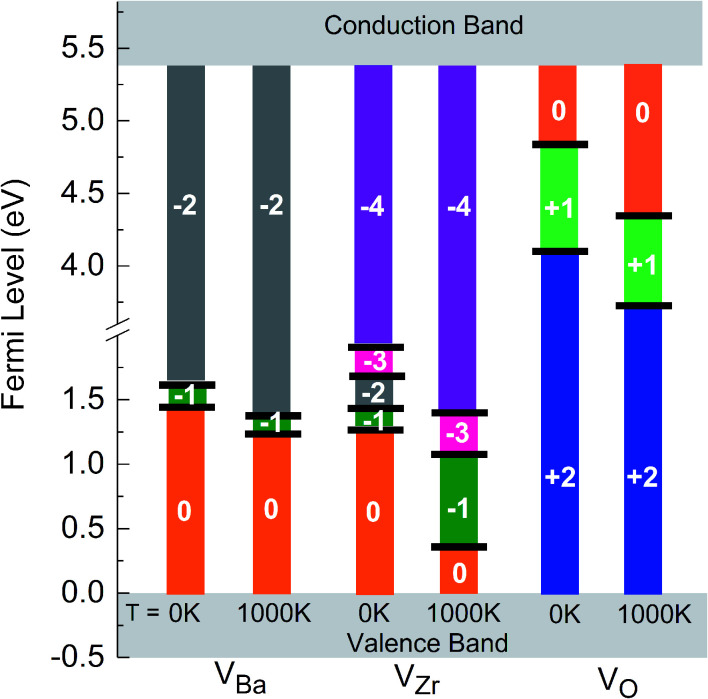
Charge transitions of Ba, Zr and O vacancies at 0 K and 1000 K.

As already mentioned above, the charge state of Ba, Zr, and O vacancies changes with increasing Fermi energy. A diagram representing all possible charge transitions of Ba, Zr and O vacancies calculated at 0 K and 1000 K is given in Fig. 6. As the Fermi level increases the Ba vacancy undergoes a charge transition from 0 to −1 to −2, whereas the Zr vacancy exhibits a transition from 0 to −1 to −2 to −3 to −4, and the O vacancy undergoes transitions from +2 to +1 to 0. At 1000 K the charge transitions occur at lower values of *E*_F_ than at 0 K. The influence of temperature on the Fermi level of the charge transition is highest for the Zr vacancy. In this case at 1000 K the range of charge state −1 is extended compared to 0 K whereas the charge state −2 is not stable so that a direct transition between −1 and −3 takes place. For O vacancies, transition levels were found closer to CBM than for the other vacancies. At 0 K the (0/+) and the (+/2+) transitions occur at a distance of 0.46 and 1.43 eV from CBM, respectively. On the other hand, at 1000 K the first and the second transition takes place at 1.15 eV and 1.77 eV, respectively, *i.e.* the range where the doubly positively charged O vacancy is stable is slightly smaller than at 0 K.

## Conclusions

A systematic DFT study on the thermodynamic stability of BZO and of Ba, Zr, and O vacancies in this material was performed. In contrast to previous investigations, which were focused on the ground state, in the present work the stability diagram of BZO was calculated for a wide range of temperatures and for different atomic reservoir conditions. With increasing temperature, the stability region shrinks. This is mainly due to the strong temperature dependence of the chemical potential of gaseous oxygen. The temperature dependence of the free formation energy of Ba, Zr, and O vacancies in BZO was determined for all possible charge states and for the different chemical environments. To the best of our knowledge, such a comprehensive analysis has not been done yet. The free formation energy of Zr vacancies depends strongly on temperature whereas this dependence is weaker for Ba and O vacancies. Accordingly, the charge transition levels are differently influenced by temperature. While at 0 K transitions between neutral, −1, −2, −3, and −4 states are found for the Zr vacancy, the −2 state disappears at 1000 K so that a direct transition between −1 and −3 takes place. Compared to 0 K at 1000 K the charge transition levels of the O vacancy are shifted towards the valence band maximum. Present first-principle investigations also indicate how an optimum proton conductivity in BZO can be achieved: the results clearly show that O poor reservoir conditions and a Fermi level close to the valence band maximum favor a high concentration of doubly positively charged O vacancies which is required to form many protonic defects. On the other hand, under these conditions the concentration of Ba and Zr vacancies is rather low so that Ba and Zr deficiencies can be avoided.

## Conflicts of interest

There are no conflicts to declare.

## Supplementary Material
